# A Split-Lung *Ex Vivo* Perfusion Model for Time- and Cost-Effective Evaluation of Therapeutic Interventions to the Human Donor Lung

**DOI:** 10.3389/ti.2024.12573

**Published:** 2024-02-28

**Authors:** Nicholas J. S. Chilvers, Jenny Gilmour, Marnie L. Brown, Lucy Bates, Chong Yun Pang, Henning Pauli, John Dark, Andrew J. Fisher

**Affiliations:** ^1^ Translational and Clinical Research Institute, Faculty of Medical Sciences, Newcastle University, Newcastle upon Tyne, United Kingdom; ^2^ Institute of Transplantation, Freeman Hospital, Newcastle upon Tyne, United Kingdom

**Keywords:** donor lungs, *ex vivo* lung perfusion, organ assessment, lung transplantation, split-lung perfusion model

## Abstract

With the ongoing shortage of donor lungs, *ex vivo* lung perfusion (EVLP) offers the opportunity for objective assessment and potential therapeutic repair of marginal organs. There is a need for robust research on EVLP interventions to increase the number of transplantable organs. The use of human lungs, which have been declined for transplant, for these studies is preferable to animal organs and is indeed essential if clinical translation is to be achieved. However, experimental human EVLP is time-consuming and expensive, limiting the rate at which promising interventions can be assessed. A split-lung EVLP model, which allows stable perfusion and ventilation of two single lungs from the same donor, offers advantages scientifically, financially and in time to yield results. Identical parallel circuits allow one to receive an intervention and the other to act as a control, removing inter-donor variation between study groups. Continuous hemodynamic and airway parameters are recorded and blood gas, perfusate, and tissue sampling are facilitated. Pulmonary edema is assessed directly using ultrasound, and indirectly using the lung tissue wet:dry ratio. Evans blue dye leaks into the tissue and can quantify vascular endothelial permeability. The split-lung *ex vivo* perfusion model offers a cost-effective, reliable platform for testing therapeutic interventions with relatively small sample sizes.

## Graphical Abstract

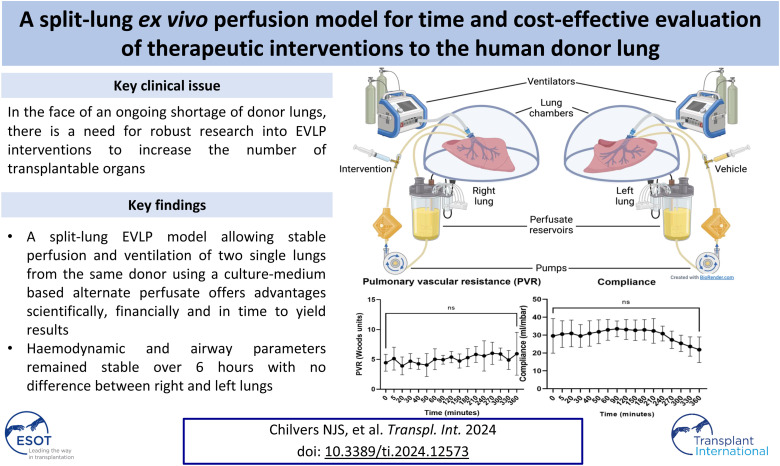

## Introduction

Despite a significant demand for organs, more than 80% of donor lungs from brain-dead donors offered for transplantation are currently declined as unsuitable in the United Kingdom [1]. This is largely because the lungs are highly susceptible to injury, which impairs function and negatively affects transplant outcomes. Marginal or “extended criteria” organs, for example, from older donors >65 years old, are rarely used. The impact for patients is that there continues to be a large discrepancy between the number of patients on the waiting list and the number of organs accepted for transplant and hence the number of lung transplants performed [1].

Research efforts have focused on preventing or minimizing donor lung injury and optimizing the quality of marginal donor organs through assessment and preservation. One such method is *ex vivo* lung perfusion (EVLP) which provides the opportunity for objective organ assessment and a time period for therapeutic repair [2, 3]. Steen et al. in Sweden performed the first clinical transplant following EVLP in 2001 using lungs from an uncontrolled Donation after Circulatory Death donor [4]. Subsequently, this group demonstrated that lungs initially rejected for transplantation could be successfully transplanted following reconditioning using EVLP [5]. Multiple studies have now shown that marginal donor lungs transplanted after EVLP have similar outcomes to standard donor organs preserved by static cold storage on ice [3, 6]. Furthermore, EVLP allows for longer preservation times, which offers logistical benefits.

EVLP was initially developed for assessment/preservation but there is increasing interest in EVLP as a platform for therapeutic intervention. This is particularly attractive as, in the isolated lung, there is no renal/hepatic excretion or risk of off-target toxicity [2]. While early research studies have focused on determining optimal perfusion parameters, further research is required to overcome the hurdles that limit wider usage and to ensure clinical translation that will maximize the potential of EVLP as a therapeutic platform [3].

In the clinical setting, EVLP is almost exclusively performed on intact double lungs, but in the research setting this approach presents challenges due to inherent variability and confounding factors between different donors in the treatment and control groups, requiring larger sample sizes. We have therefore developed a split-lung human EVLP model consisting of two identical independent perfusion circuits, each with its own ventilator. Recently mechanisms of IL-1β-mediated Inflammation [7]. Since then, we have further optimized this setup, including more comprehensive pressure monitoring, and tested Dulbecco’s Modified Eagle Medium (DMEM)-based perfusate, previously described by Shaver et al. [8]. This model offers a cost-effective, reliable platform for testing therapeutic interventions with relatively small sample sizes.

## Materials and Methods

### Research Approval and Ethics

Donor organs, declined for transplantation, are only utilised where consent has been obtained for research by Specialist Nurses in Organ Donation. The NIHR Blood and Transplant Research Unit laboratory in Newcastle has ethical approval from the NHS North East Research Ethics Committee, reference 16/NE/0230, and NHSBT (Study 66).

### Donor Lung Retrieval and Preparation

The UK benefits from the national “Increasing the Number of Organs Available for Research” (INOAR) framework, established by National Health Service Blood and Transplant (NHSBT), through which the next of kin of organ donors are approached for consent for research if organs are not suitable for transplantation. Patient history, clinical findings, and diagnostic investigations, including chest imaging, are reviewed to assess suitability for EVLP studies. Due to our split-lung model, we excluded lungs with unilateral pathology, e.g., trauma, infection, or consolidation, in addition to lungs with significant trauma/contusions, blood-borne viruses, or significant/untreated infection. Donor lungs were assessed and explanted by the NHSBT National Organ Retrieval Service Cardiothoracic Teams in a standard fashion, including antegrade and retrograde flushing with Perfadex (XVIVO Perfusion AB, Sweden) and inflation with 50% O_2_, prior to static cold storage in an icebox. Certified medical couriers transported the lungs from the donor hospital to our research laboratory.

**FIGURE 1:**
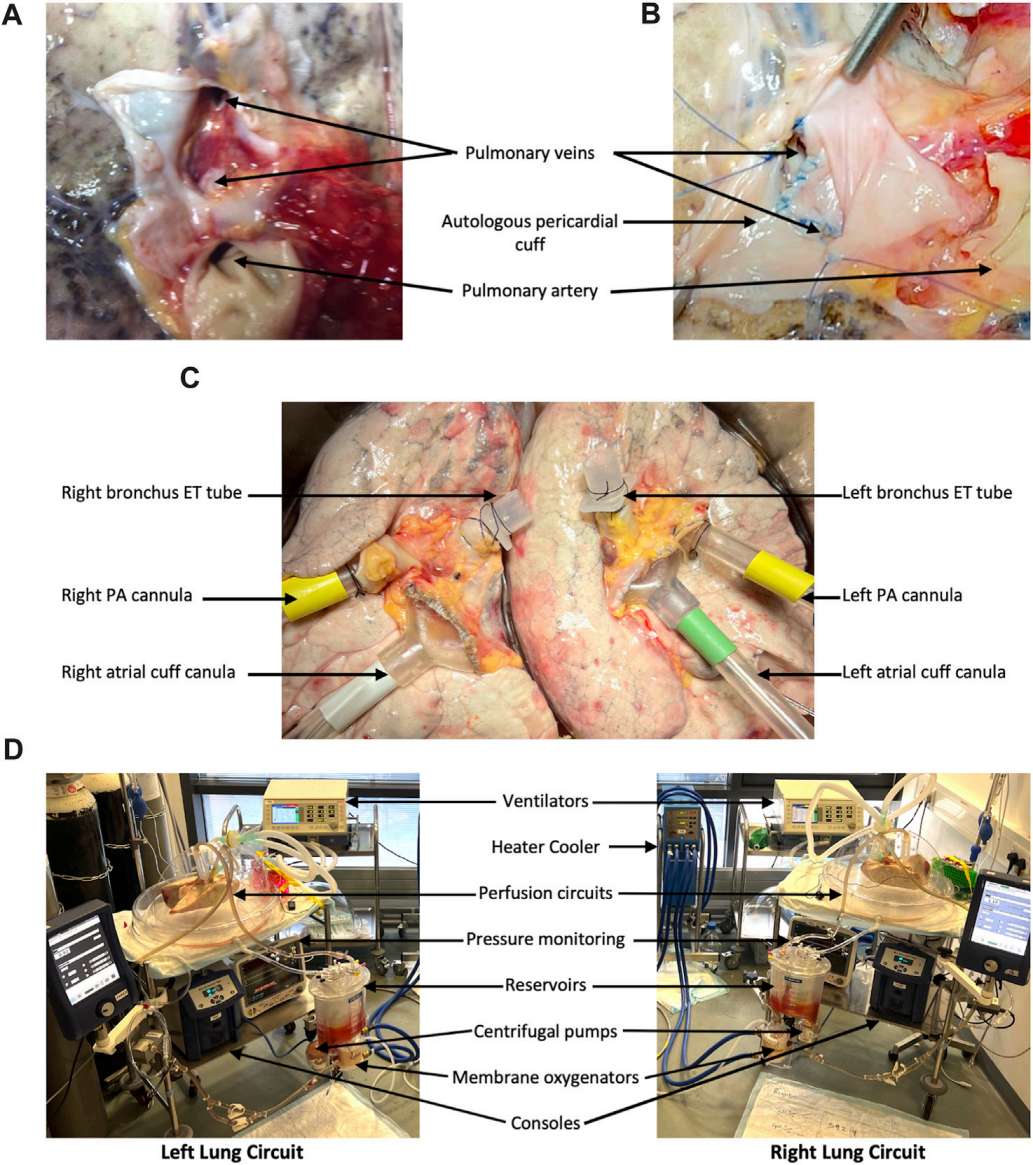
Lung preparation and split-lung circuits. In order to have a closed left atrium (LA) in the circuit, donor lungs without a sufficient LA cuff **(A)** require reconstruction with autologous pericardium **(B)**. The lungs are separated before EVLP cannulae are sutured to the LAs and pulmonary arteries (PAs) and shortened ET tubes are secured in the left and right main bronchi **(C)**. Our split-lung model consists of two separate identical circuits attached to two ventilators **(D)**.

Upon arrival in the laboratory, the lungs were placed in cold 0.9% saline. Excess tissue and pericardium were excised and dissection was carried out to prepare the pulmonary arteries, veins, and bronchi for cannulation/intubation. The posterior wall of the left atrium (LA) was divided in the midline to create two LA cuffs, left and right, each receiving a superior and inferior pulmonary vein. On occasion, the received lungs may have had very little LA tissue (Figure 1A) and the cuffs had to be reconstructed with autologous pericardium (Figure 1B). Two XVIVO EVLP LA cannulae (XVIVO Perfusion AB, Sweden) were cut to size and sutured to the LA cuffs using 4-0 Prolene (Figure 1C). The pulmonary artery (PA) was divided at its bifurcation and two XVIVO PA cannulae were inserted into the left and right PAs and secured with 2-0 silk purse-string sutures (Figure 1C). Finally, the left main bronchus was clamped and divided proximally (keeping, initially, the left lung inflated). This bronchial stump was then securely closed, flush with the trachea, allowing separate intubation of the main trachea (well clear of the right upper lobe orifice) and then separately of the left main bronchus. The endotracheal tubes were secured with 2-0 silk sutures (Figure 1C).

### DMEM Perfusate

A 5% bovine serum albumin (BSA) solution was prepared by adding 25 g of BSA (Melford Laboratories Ltd., United Kingdom) to each 500 mL of DMEM-high glucose, without phenol red (Sigma-Aldrich, United States). The solution was mixed with a magnetic stirrer until dissolved before purification using a vacuum filter unit. DMEM perfusate was stored at 4° until required.

### EVLP

The EVLP setup (Figure 1D) and protocol for this model are based on those described in the Toronto protocol [9]. Two separate but identical circuits (Medtronic Limited, United Kingdom) consisted of outflow tubing to a reservoir from which perfusate was pumped, by a centrifugal pump, through a membrane oxygenator (attached to a heater-cooler) to the inflow tubing. Circuits were primed with 1500 mL of perfusate and 7500 IU of heparin and heated initially to 32°. Cardiac output was calculated based on the ideal donor body weight and divided between the left and right lungs with a 45%/55% split. Flow was started at 20% and the PA cannulae were attached to the lungs followed by the LA cannulae. Pressure monitoring was attached to the PA and LA cannulae. The LA pressure was maintained between 3 and 5 mmHg by adjusting a gate clamp on the outflow tubing, the LA pressure was maintained between 3 and 5 mmHg. Over 15 min, flow was increased to 40%. Once the lung temperature reached 32°, two separate ventilators were attached to the ET tubes. The protective ventilation protocol (Supplementary Table S1) slowly increased tidal volume with each further degree of temperature increase until tidal volumes of 7 mL/kg (split 45%/55%) were reached. Regular blood gas analyses were carried out. CO_2_ flow to the membrane oxygenator was adjusted to maintain pCO_2_ between 3 and 5 kPa. Tris (hydroxymethyl)aminomethane was added to maintain a pH between 7.35 and 7.45.

### Assessment of Lung Physiology

Hemodynamic parameters including PA pressures and ventilation parameters such as compliance and peak airway pressures were measured continuously throughout the perfusion. Regular blood gas and perfusate samples were taken according to a comprehensive established protocol. Depending on the research question, tissue samples were also collected for histological and transcriptomic analysis.

### Assessment of Pulmonary Vascular Leak

Pulmonary edema/extravascular lung water were assessed by lung weight, tissue sampling for wet-to-dry ratio, and ultrasound assessment using the DireCt Lung Ultrasound Evaluation (CLUE) score [10]. Additionally, after 2 h of perfusion, 0.05% Evans blue (0.75 g/1500 mL) was added to each circuit, and tissue and bronchioalveolar lavage (BAL) samples were taken 2 and 4 h later. Evans blue concentration is measured spectrophotometrically as a marker of endothelial permeability.

### Statistics

Data are presented as mean ± standard deviation (SD). Weight increase was compared using a Student’s unpaired *t*-test, whereas one-/two-way repeated measures ANOVAs or mixed-effects analyses with multiple comparisons were used for hemodynamic/ventilation parameters. Evans blue concentration was analyzed using a Student’s paired t-test or one-way ANOVA. Statistical tests were performed using GraphPad Prism 9.5.0 and *p* values less than 0.05 were considered statistically significant.

## Results

### The Split-Lung Model With DMEM-Based Perfusate Provides Stable Perfusion for 6 h

**FIGURE 2:**
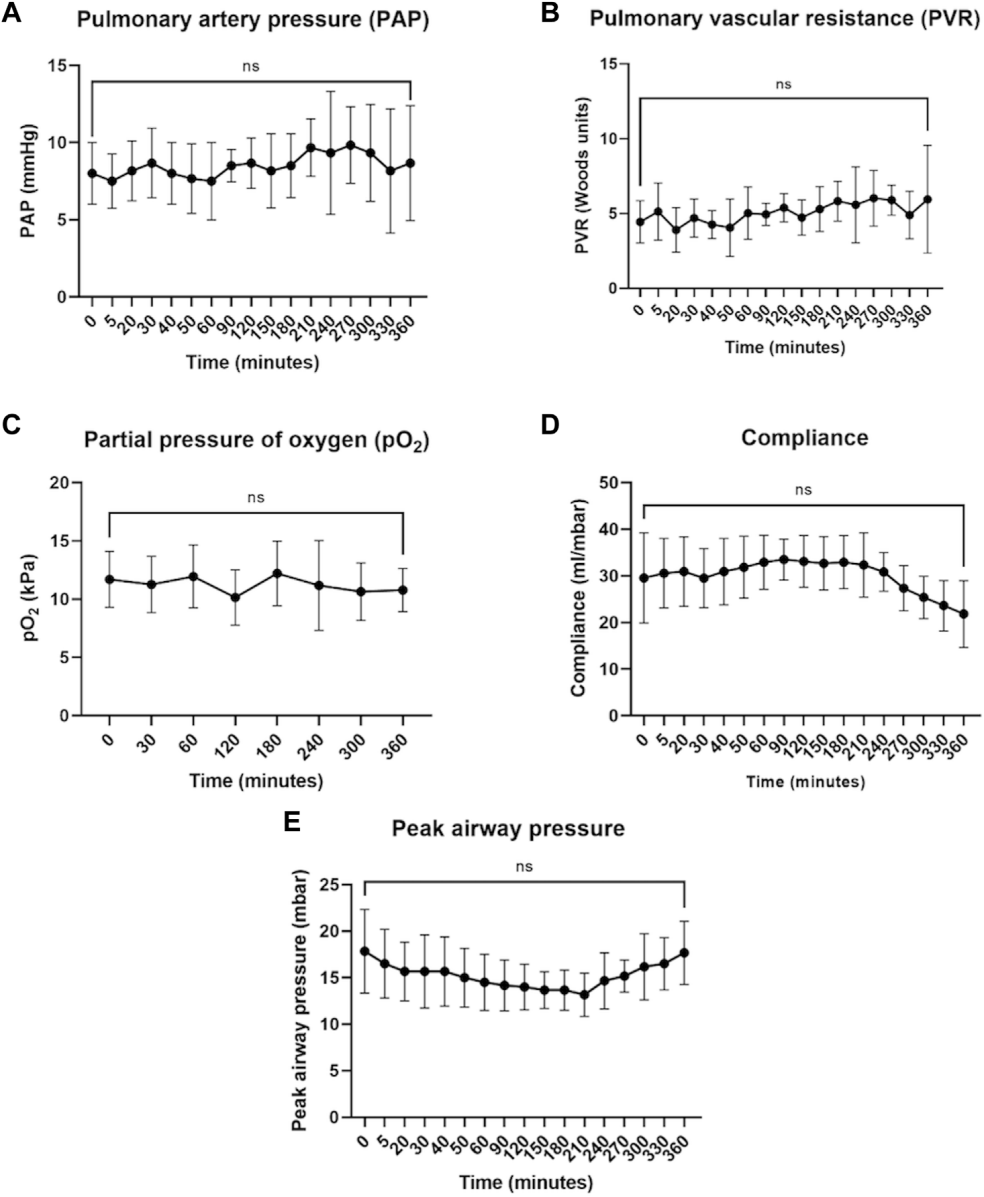
Hemodynamic, blood gas, and airway parameters during 6 h of perfusion. Pulmonary artery pressure **(A)**, pulmonary vascular resistance **(B)**, and partial pressure of oxygen **(C)** remained stable throughout perfusion. Compliance **(D)** and peak airway pressure **(E)** showed some improvement but by the end of 6 h returned to values similar to time 0 (defined as the point at which full flow and ventilation were established). The data are expressed as mean ± SD, and analyzed with one-way repeated measures ANOVA and with Dunnett’s multiple comparisons test to determine statistical significance compared to time 0. The data presented are *n* = 6 unpaired control lungs from six independent split-lung perfusions.

Lungs were retrieved from 6 donors (5 men and 1 woman, 1 DCD donor, and 5 DBD donors) with a mean age of 34.8 ± 10.2 years. The cause of death was either hypoxic brain damage or intracranial hemorrhage and the lungs were rejected for clinical transplantation due to poor function (*n* = 3), lack of suitable recipients (*n* = 2), or infection (*n* = 1). During 6 h of normothermic perfusion of control single lungs with prolonged cold ischemic times (11.5 ± 1.8 h), there was no significant change in hemodynamic parameters, including pulmonary artery pressure (Figure 2A) and pulmonary vascular resistance (Figure 2B), blood gas parameters such as pO_2_ (Figure 2C) and compliance (Figure 2D). Peak airway pressure varied over time (ANOVA *p* = 0.02), but multiple comparisons were not significant. At 6 h there was no significant difference from time 0 (Figure 2E). There was no difference in parameters between the left and right lungs (Supplementary Figure S1).

In a study of four unpaired single lungs comparing DMEM versus STEEN solution as a perfusate, we found no significant difference in the percentage increase in lung weight (46.7% ± 23.2% vs. 99.4% ± 36.3%, *p* = 0.226).

### The Split-Lung Model Provides a Platform for Comprehensive Lung Assessment

**FIGURE 3:**
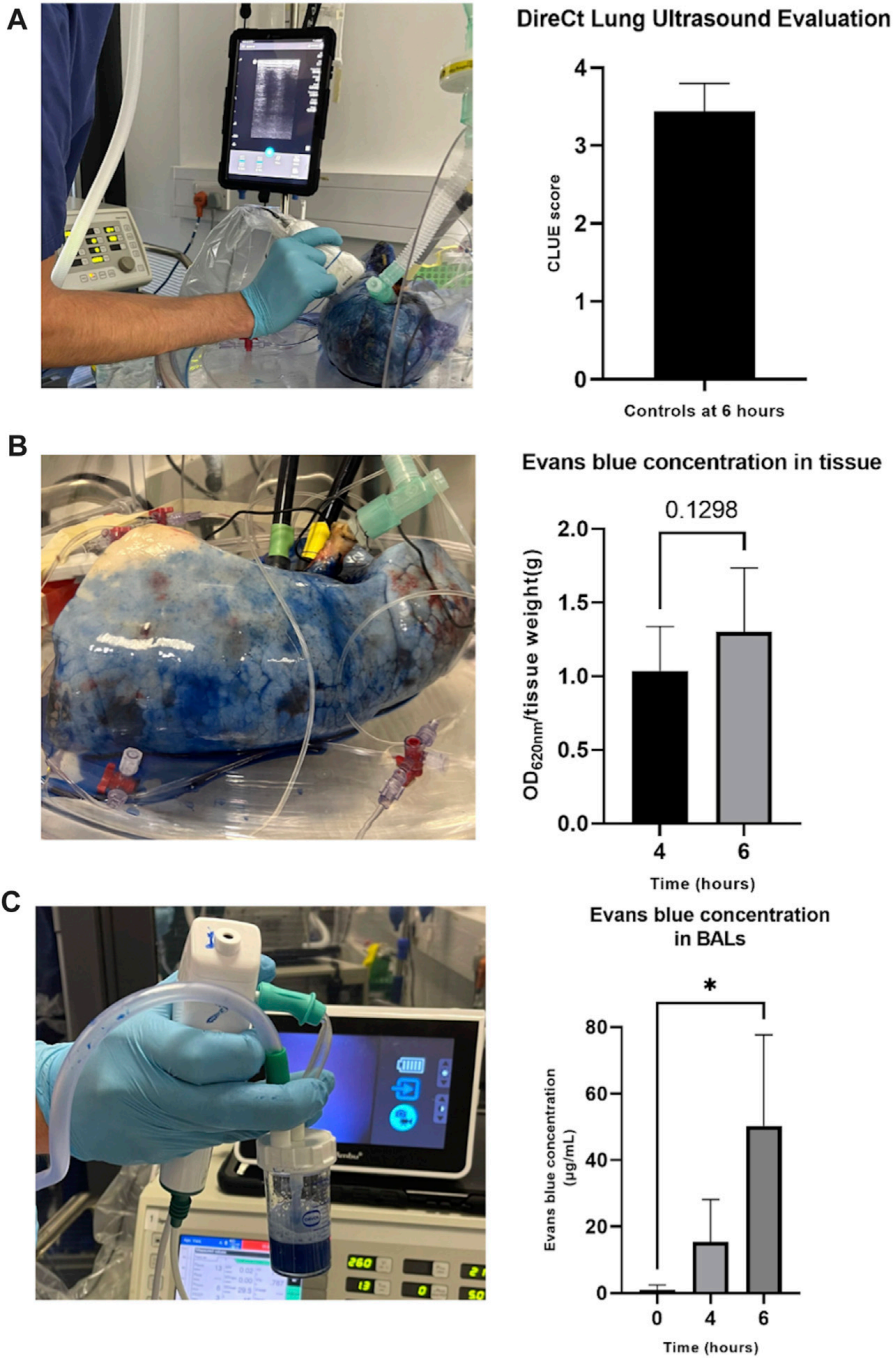
Assessment of pulmonary edema/extravascular lung water during perfusion. DireCt Lung Ultrasound Evaluation (CLUE) was performed by using ultrasound to assess 14 (left) or 16 (right) areas of the lung, providing an average CLUE score **(A)**. Evans blue concentration in tissue was analyzed by placing biopsy samples in formamide and heating at 55°C for 24 h before centrifuging, transferring supernatant samples to a 96-well plate, and reading using a spectrophotometer at 620 nm **(B)**. Evans blue concentration was measured in BAL samples by centrifugation, placing the supernatant in a 96-well plate and reading alongside a standard curve on the spectrophotometer at 620 nm **(C)**. The data are expressed as mean ± SD, and analyzed by a Student’s paired t-test **(B)** or one-way repeated measures ANOVA and Dunnett’s multiple comparison test **(C)**. The data presented are *n* = 6 unpaired control lungs from six independent split-lung perfusions. *p < 0.05.

Pulmonary edema/extravascular lung water could be readily evaluated using several techniques. The ultrasound CLUE score uses a non-invasive technique to quantify edema (Figure 3A). Evans blue could be appreciated visibly, and quantified at multiple time points, from both tissue (Figure 3B) and BAL (Figure 3C) samples. Both increased during perfusion, reaching significance in BALs. Additionally, lung weights were measured pre- and post-perfusion and a large tissue sample was taken to calculate a wet-to-dry ratio (data not shown).

### Split-Lung Perfusion With DMEM-Based Perfusate Offers a Cost-Effective Research Model

Table 1 displays the costs of consumables for the 2 separate circuits that comprise the split-lung model. Additional costs, not shown, include staff time, specialized courier transport of the organs, and equipment rental/maintenance.

**TABLE 1:** Costs of consumables for the split-lung model (2 identical circuits) and DMEM-based perfusate. Costs are based on quotes that were correct at the time of manuscript submission.

Consumables	DMEM perfusate protocol
Lung perfusion Circuits × 2	£1,200
Lung cannula set	£705
Perfusate (4 L)	
* *DMEM	£56
* *Bovine Serum Albumin	£60
* *Disposable bottle top filters	£28
* *Heparin	£4
Plasticware and consumables	£200
* *Ventilator consumables, suction consumables, pressure monitoring lines, sutures, scalpels, staples, specimen tubes, cryovials	
Medical gas/air	£45
Blood gas analyzer cartridges/consumables	£110
Reagents	£65
* *Formalin, RNA later, THAM, formamide, Evans blue	
Total cost	£2,473

The cost of 4 L of DMEM-based perfusate is £148, which is significantly less expensive than more commonly used options such as STEEN solution. As a result, the total cost of consumables, approximately £2,473/€2879/$3,107, makes this approach affordable in the research setting.

## Discussion

There is a growing interest in the optimization and assessment of extended criteria donor lungs using EVLP. Research initially focused on establishing optimal perfusion protocols, parameters for functional lung assessment, and, in clinical studies, their impact on, or correlation with, patient outcomes [2, 3]. Recent studies have proposed more accurate methods of assessment, including the CLUE score [10] and the “PaO_2_/FiO_2_ ratio difference” (the difference between PaO_2_/FiO_2_ at a FiO_2_ of 1.0 and 0.4) [11]. Lately, research has shifted focus to more sophisticated biomarkers and to assessing the efficacy of interventions on lung function or in reducing risks after implantation. EVLP is particularly attractive for the latter due to the absence of off-target effects [2]. Studies have also suggested that it may even be possible to treat transmissible conditions from the donor, such as hepatitis C and cytomegalovirus [12, 13].

We have described an optimized split-lung EVLP model based on the Toronto perfusion protocol [9], which is generally accepted as being preferable for longer preservation studies [14]. Acellular perfusate removes the risk of infection and hemolysis, simplifies logistics, and has been shown to have no detrimental effect on physiological outcomes compared to cellular perfusate [15, 16]. The lower flow rates are associated with improved oxygenation [14, 17] and wet-to-dry ratio [17]. A closed LA offers improved hemodynamic parameters, compliance and oxygenation, and less edema [18]. Our 45%/55% split of donor-specific calculated flow and tidal volumes ensures comparable results between the left and right lungs. Accurate pressure monitoring in both LAs and PAs allows the maintenance of physiological conditions. Recently, we have introduced DMEM-based perfusate [8], although blood or specific cells such as isolated neutrophils could be added depending on the research question. Huang et al. showed that a similar DMEM-based perfusate reduced apoptosis and increased both glutathione and heat shock protein 70 protein levels compared to STEEN, in an EVLP cell culture model [19]. DMEM also has a higher osmolality (317–351 mOsm/kg vs. 275–315 mOsm/kg).

The major benefit of the split-lung model is the intra-donor control. This allows research studies to use smaller numbers of replicates per experiment as each lung pair comes with a treatment and control organ. This is especially important in human research because lungs are such a precious resource, but most importantly it ensures that they are better matched, eliminating the numerous inherent confounding factors of having entirely different donors in the treatment and control groups. The advantages of this approach have been described in the context of hepatitis C treatment trials, as the control lung would have a similar viral load [12]. This methodology has been used in our laboratory to understand the mechanism of interleukin-1β driven inflammation during EVLP [7], and to assess the therapeutic effects of endothelial barrier protection, potential COVID-19 treatments [20], cell therapies, and, currently, extracellular vesicles, with significant results from only five lung pairs. Smaller numbers allow studies to be completed more quickly and mitigate the risk of unbalanced groups in the event of recruitment failure, as both arms are balanced throughout. This model could also be used for large animal research where an experimental transplantation outcome is required as part of the investigation.

The affordability of this set-up, largely due to the modest costs of DMEM perfusate, makes it a more viable research model. The smaller number of experimental replicates more than offsets the cost of the additional consumables per lung pairs through savings in organ transport and personnel costs. Furthermore, cannulae and lung domes can be reused multiple times for pre-clinical research so the costs listed in Table 1 are overestimates. Strong links through collaborative work between our university laboratory and the hospital provide access to equipment such as ICU standard ventilators retired from clinical service, and expired consumables.

To improve organ utilization, several countries, including the United Kingdom, have either established or are actively considering a system of centralized organ “Assessment and Recovery Centres.” Benefits include centralized expertise, as lower-volume centers have been observed to have worse outcomes [21], standardization of protocols, and the ability to conduct multicenter trials. A feasibility study in the US demonstrated similar survival rates following transplantation using organs from a centralized EVLP program compared to conventional lung transplant recipients [22]. The authors suggest that centralization will be particularly important as EVLP is increasingly used for the delivery of therapeutics especially if prolonged perfusion is required to facilitate this. There is therefore a need for concurrent research into accurate markers of organ injury/function and potential therapeutics. Organ assessment and repair centers increase the opportunities for both clinical and preclinical research, as organs turned down before or during clinical EVLP can also be used in preclinical studies.

### Considerations/Limitations

Although affordable, there are still significant costs and complex logistics associated with this model. We mitigate the former somewhat by not priming circuits until we have assessed the donor lung pairs on arrival. The split-lung closed LA model requires greater surgical expertise for timely cannulation, particularly if reconstruction is required. Finally, the lung weight data presented here for STEEN vs. DMEM were from unpaired lungs and were not adjusted for donor differences. However, we were satisfied with the performance of DMEM and, in the research setting, STEEN is not a feasible expense for most research centers.

## Conclusion

In an exciting era where centralized lung assessment and repair may become the norm, this split-lung EVLP model, using a culture medium-based alternate perfusate, offers a cost-effective way to test therapeutic interventions with relatively small sample sizes. The in-built control offered by the contralateral lung affords robust results and we would encourage the adoption of this model for future preclinical EVLP research.

## Data Availability

The raw data supporting the conclusion of this article will be made available by the authors, without undue reservation.
