# Supplementary figures and images for: A Split-Lung Ex Vivo Perfusion Model for Time- and Cost-Effective Evaluation of Therapeutic Interventions to the Human Donor Lung
Source: Transpl Int. 2024 Feb 28;37:12573. doi: 10.3389/ti.2024.12573 (PMC10933070; doi:10.3389/ti.2024.12573)

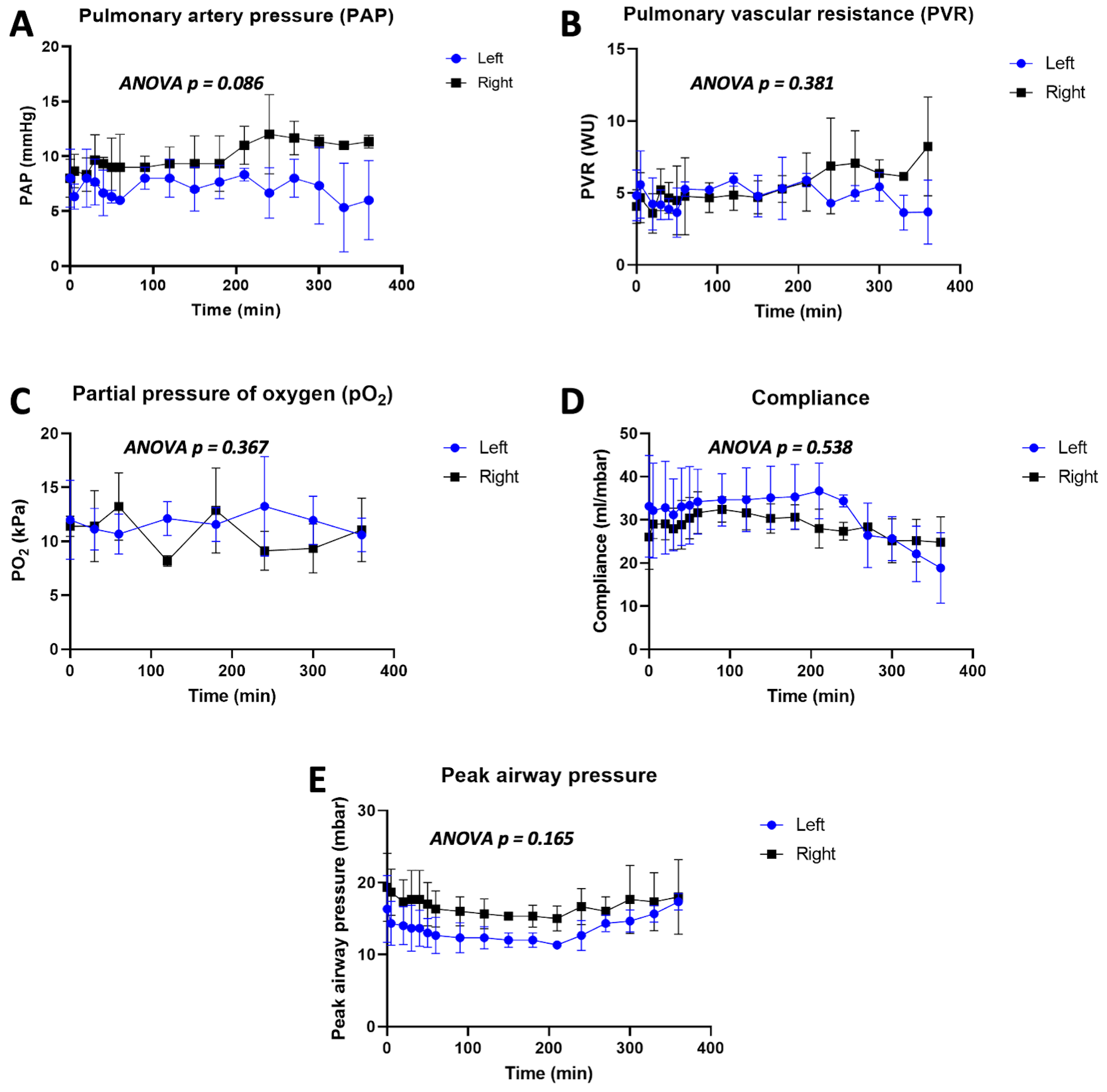

Supplement: Supplementary file 1 [file Image1.TIFF]
