# Supplementary material for: A Split-Lung Ex Vivo Perfusion Model for Time- and Cost-Effective Evaluation of Therapeutic Interventions to the Human Donor Lung
Source: Transpl Int. 2024 Feb 28;37:12573. doi: 10.3389/ti.2024.12573 (PMC10933070; doi:10.3389/ti.2024.12573)
Supplement: Supplementary file 3 [file DataSheet1.docx]

**Supplementary data and legends**

**Supplementary Table 1 – Protective ventilation protocol for split-lung perfusion with two independent ventilators.** Tidal volumes are calculated based on donor ideal body weight and split between left and right lungs 45%/55%. With each degree increase in lung temperature, tidal volumes and/or respiratory rate are increased until the maximum target volume (6-7ml/kg split between both lungs) is achieved.

**Supplementary Figure 1 – Comparison of haemodynamic, blood gas and airway parameters between left and right lungs during 6 hours of perfusion.** Pulmonary artery pressure (A), pulmonary vascular resistance (B) and partial pressure of oxygen (C), Compliance (D) and peak airway pressure (E) showed no significant differences between left and right lungs during 6 hours of perfusion. Data expressed as mean ± SD, and analysed with two-way repeated measures ANOVA (A,B,D,E) or mixed-effects analysis (C; due to a missed blood gas sample) with Dunnett’s multiple comparisons to determine statistical significance between left and right lungs. Data presented is n=6 unpaired control lungs from 6 independent split-lung perfusions. *p <0.05.
